# Hydrogen Sulfide Protects Against Cerebral Ischemia–Reperfusion Injury in Rats via S-Sulfhydrating NAMPT to Enhance Mitochondrial Function and Autophagy in Cerebrovascular Endothelial Cells

**DOI:** 10.3390/ph19050742

**Published:** 2026-05-08

**Authors:** La Jiang, Shuai Liang, Yu Jiang, Jia-Rong Jiang, Shan Wang, Xiaojiao Yin, Zhiwu Chen, Ji-Yue Wen, Shuo Chen

**Affiliations:** 1 Department of Pharmacology, School of Pharmaceutical Sciences, Anhui Medical University, Hefei 230032, China; 2346010032@stu.ahmu.edu.cn (L.J.); 2345010187@stu.ahmu.edu.cn (S.L.); 2001500028@ahum.edu.cn (J.-Y.W.); 2 Clinical Medical College, Anhui Medical University, Hefei 230012, China; 3 Key Laboratory of Xin’An Medicine, Ministry of Education, Anhui University of Chinese Medicine, Hefei 230038, China

**Keywords:** hydrogen sulfide, nicotinamide phosphoribosyltransferase, S-sulfhydration, mitochondrial function, cerebrovascular endothelial cells, ischemic stroke

## Abstract

**Objective:** Cerebral ischemia–reperfusion (I/R) injury constitutes a pivotal pathological driver in cerebrovascular disorders such as stroke, yet effective therapeutic interventions remain scarce. This study explored whether hydrogen sulfide (H_2_S) mitigates endothelial cell damage in the cerebral vasculature during I/R by modulating nicotinamide phosphoribosyltransferase (NAMPT) activity and its S-sulfhydration status, consequently restoring mitochondrial integrity and energetic homeostasis. **Methods:** Primary cerebrovascular endothelial cells (ECs) were subjected to hypoxia/reoxygenation (H/R) conditions in vitro, while rats experienced middle cerebral artery occlusion/reperfusion (MCAO/R) in vivo. The H_2_S donor sodium hydrosulfide (NaHS) was administered, and outcomes were evaluated through Western blot analysis, S-sulfhydration assays, mitochondrial functional tests, autophagy profiling, and neurobehavioral assessments. The contributions of NAMPT and S-sulfhydration were validated using FK866 and dithiothreitol (DTT), respectively. LC-MS/MS was employed to identify candidate S-sulfhydration sites on NAMPT triggered by H_2_S. **Results:** In cellular models, NaHS substantially boosted NAMPT enzymatic activity, elevated NAD^+^ and ATP levels, and enhanced cell survival. These protective benefits were nullified upon NAMPT inhibition with FK866 or reversal of S-sulfhydration via DTT. In animal studies, NaHS treatment significantly diminished infarct volume and ameliorated neurological deficits in MCAO/R rats; however, pretreatment with FK866 or DTT attenuated these benefits. Mechanistic investigations revealed that NaHS promoted S-sulfhydration of NAMPT, thereby activating autophagy of dysfunctional mitochondria. LC-MS/MS analysis confirmed enhanced S-sulfhydration at Cys39 and Cys397 residues of NAMPT following H_2_S exposure. **Conclusions:** H_2_S exerts neuroprotection against cerebral I/R injury in rats through S-sulfhydration-mediated activation of NAMPT, which improves mitochondrial performance and stimulates autophagy in cerebrovascular ECs.

## 1. Introduction

Ischemic stroke, frequently referred to simply as “stroke,” stands among the foremost causes of disability and death worldwide [1]. Its core pathological mechanism involves reperfusion injury following cerebral ischemia—a process wherein the restoration of blood flow, while attempting to salvage threatened brain tissue, exacerbates oxidative stress, inflammatory responses, and cellular energy crisis [2,3,4]. This cascade ultimately leads to blood–brain barrier disruption and neuronal death [5]. Currently, treatment options targeting this complex pathological process remain limited, underscoring an urgent need for novel neuroprotective strategies with clinical translational potential.

In recent years, endogenous gasotransmitters, especially hydrogen sulfide (H_2_S), have attracted considerable interest due to their demonstrated protective roles in various neurological conditions [6]. Exogenous supplementation with hydrogen sulfide (H_2_S) or its donors has been demonstrated to exert potent antioxidant, anti-inflammatory, and anti-apoptotic activities across diverse experimental models of brain injury [7,8]. Nevertheless, the specific molecular targets and downstream signaling cascades mediating these cytoprotective actions remain poorly defined, thereby limiting the full therapeutic realization of H_2_S.

NAMPT functions as the rate-limiting enzyme within the mammalian NAD^+^ salvage biosynthesis pathway [9,10]. As an essential cofactor in numerous redox reactions, NAD^+^ constitutes a metabolic cornerstone for mitochondrial respiratory chain function, DNA repair, and the activity of sirtuin family proteins [11,12]. Under ischemic stress, NAD^+^ levels decline precipitously, resulting in impaired ATP production and mitochondrial dysfunction—key drivers of cellular demise [13,14,15]. Consequently, maintaining or enhancing NAMPT activity to stabilize NAD^+^ pools has emerged as a promising neuroprotective strategy. Notably, protein S-sulfhydration—a reversible post-translational modification mediated by H_2_S, analogous to phosphorylation—regulates protein activity, localization, and interactions via the addition of sulfhydryl groups to cysteine residues [16,17,18]. Given the biological effects of H_2_S and the central role of the NAMPT/NAD^+^ axis in cellular energy homeostasis, we hypothesize that H_2_S may enhance NAMPT enzymatic activity through S-sulfhydration of critical cysteine residues, thereby representing a core mechanism underlying its protective role on cerebrovascular ECs and mitochondrial function.

Cerebrovascular ECs constitute the structural and functional basis of the blood–brain barrier [19]. As the first line of defense against ischemic reperfusion injury, their dysfunction significantly exacerbates cerebral edema and neuroinflammation [20,21]. However, whether and how H_2_S directly regulates NAMPT via post-translational modification to influence the NAD^+^ salvage pathway in cerebrovascular endothelial cells during I/R injury remains entirely unknown. Here, we report for the first time that H_2_S exerts its cerebrovascular protective effects by S-sulfhydrating NAMPT, thereby preserving its enzymatic activity, maintaining NAD^+^ homeostasis, and protecting mitochondrial function. And we report for the first time that H_2_S significantly enhances S-sulfhydration of NAMPT at Cys39 and Cys397. This H_2_S-NAMPT-NAD^+^–mitochondria signaling axis represents a novel mechanistic insight into the endogenous defense against cerebral I/R injury. Therefore, this study focuses primarily on cerebrovascular ECs, aiming to systematically investigate whether H_2_S modulates intracellular NAD^+^/ATP levels via NAMPT S-sulfhydration, thereby improving mitochondrial function—including membrane potential, reactive oxygen species (ROS) production, and calcium homeostasis—and regulating autophagic activity. Ultimately, this work seeks to elucidate how H_2_S mitigates damage in models of ischemic stroke simulated by H/R and MCAO/R. A multidisciplinary approach incorporating molecular biology, cellular metabolomics, and animal behavioral assays will be employed.

In summary, our study identifies NAMPT as a novel functional target of H_2_S in cerebrovascular endothelial cells. We demonstrate that H_2_S-mediated S-sulfhydration of NAMPT is a critical mechanism that sustains NAD^+^ biosynthesis and mitochondrial integrity during I/R injury. And H_2_S significantly enhances S-sulfhydration of NAMPT at Cys39 and Cys397 in vitro. These findings elucidate a previously unrecognized H_2_S-NAMPT-NAD^+^ signaling axis in cerebrovascular protection, offering a potential new therapeutic avenue for ischemic stroke.

## 2. Results

### 2.1. Protection of NaHS Against H/R Injury in Rat Cerebrovascular ECs, and Its Effect on the NAMPT Activity and Productions of NAD^+^ and ATP

**Figure 1:**
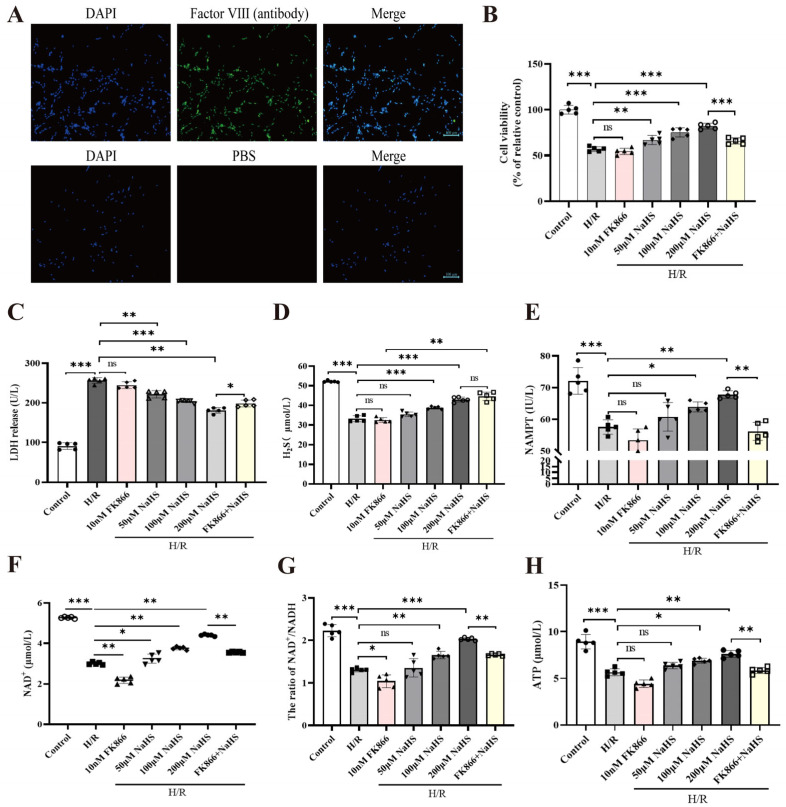
Impact of NAMPT inhibition on H_2_S-mediated alleviation of H/R injury in rat cerebrovascular ECs. (**A**) Characterization of primary rat cerebrovascular ECs via immunofluorescence using anti-factor VIII antibody (green cytoplasmic signal); nuclei were counterstained with DAPI (blue). Scale bar: 100 μm. (**B**) EC viability. (**C**–**H**) Assessments of LDH release, H_2_S concentration, NAMPT activity, NAD^+^ levels, NAD^+^/NADH ratio, and ATP content. Statistical significance: * *p* < 0.05, ** *p* < 0.01, *** *p* < 0.001 versus indicated groups; ns, no significance. Data are expressed as mean ± SD (n = 5; one-way ANOVA).

As illustrated in Figure 1A, nuclei of cultured rat cerebrovascular cells exhibited blue fluorescence following DAPI staining, while cell bodies displayed green immunofluorescence upon labeling with an antibody against the endothelial-specific marker factor VIII; no such signal was detected in PBS-treated controls, confirming the identity of the cultured cells as rat cerebrovascular ECs. Figure 1B through Figure 1D reveal that treatment with the H_2_S donor NaHS at concentrations of 50, 100, and 200 μM significantly attenuated H/R-induced reductions in EC viability and endogenous H_2_S levels, while concurrently suppressing the elevation of lactate dehydrogenase (LDH) release. These results collectively demonstrate that H_2_S confers substantial protective benefits against H/R-mediated cellular injury.

In rat cerebrovascular ECs, data presented in Figure 1B–D reveal that FK866 (10 nM), a specific NAMPT inhibitor, exerted no influence on the H/R injury-induced decline in H_2_S levels and cell viability, nor on the surge in LDH release. However, FK866 significantly reversed the protective role conferred by NaHS (200 μM) on these parameters, implying that NAMPT mediates the cytoprotective action of H_2_S against H/R insult in rat cerebrovascular ECs.

Furthermore, NaHS administration at concentrations of 50, 100, and 200 μM potently counteracted the H/R-triggered suppression of NAMPT activity and NAD^+^ levels; conversely, co-treatment with FK866 (10 nM) markedly blunted the restorative impact of 200 μM NaHS (Figure 1E,F). Given that the NAD^+^ salvage pathway constitutes the primary route for cellular NAD^+^ biosynthesis, with NAMPT serving as its rate-limiting enzyme, these findings suggest that H_2_S facilitates NAD^+^ generation by upregulating NAMPT in rat cerebrovascular ECs.

Nicotinamide adenine dinucleotide (NAD), or coenzyme I, exists in oxidized (NAD^+^) and reduced (NADH) forms, playing pivotal roles in energy metabolism, including glycolysis, fatty acid oxidation, and the tricarboxylic acid cycle. Moreover, Figure 1G,H illustrate that NaHS (50, 100, and 200 μM) substantially mitigated the H/R-induced reductions in both the NAD^+^/NADH ratio and ATP content, effects that were significantly abolished by FK866. Collectively, these results not only corroborate that H_2_S boosts NAD^+^ production via NAMPT but also indicate that H_2_S enhances ATP synthesis through the NAMPT-NAD^+^ axis. This aligns with the established role of NAMPT in NAD^+^ biosynthesis and the known bioenergetic functions of NAD^+^, while extending current understanding by directly linking H_2_S to ATP synthesis through the NAMPT-NAD^+^ axis.

### 2.2. Effect of FK866 on the Protective Effect of NaHS Against Cerebral I/R Injury in Rats

**Figure 2:**
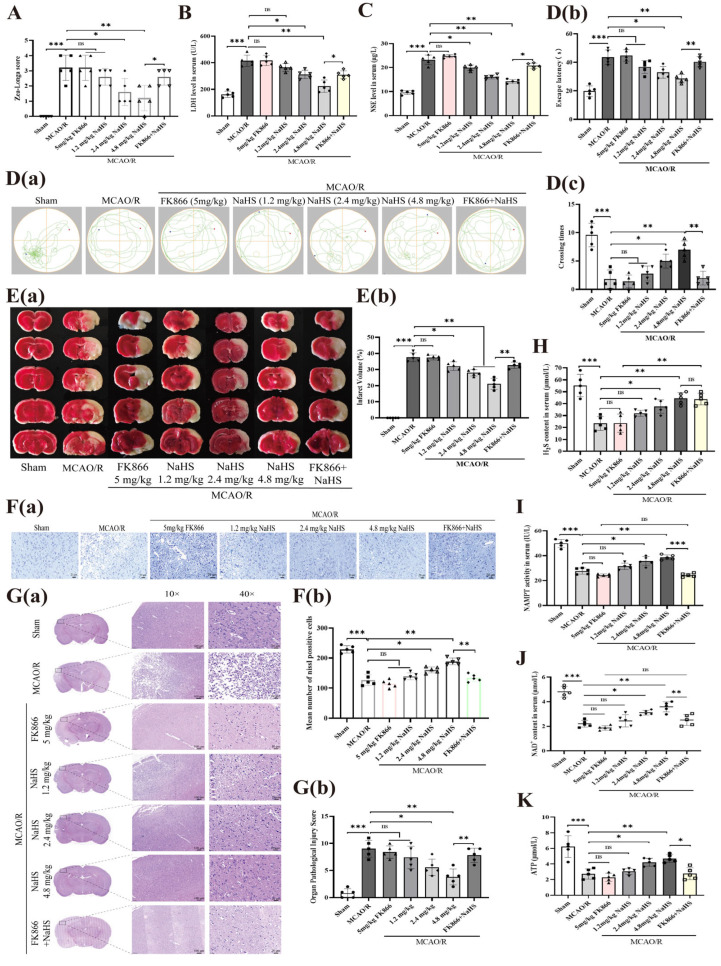
Impact of NAMPT inhibition on the ameliorative effects of H_2_S against cerebral I/R injury in rats. (**A**) Assessment of neurological deficits via Zea-Longa scoring following MCAO/R. (**B**,**C**) Serum levels of LDH and NSE. (**D**) Morris water maze performance, including statistical analysis of escape latency and platform crossing frequency; (**a**) Representative diagram of swimming trajectories of rats in each group; (**b**) Comparison of escape latency among different groups of rats; (**c**) Comparison of the number of times each group of rats crosses the platform. (**E**) (**a**) Representative TTC-stained brain sections and (**b**) quantitative analysis of infarct volume across groups. (**F**) (**a**) Nissl staining images with (**b**) quantification of Nissl-positive cells (scale bar: 20 μm). (**G**) (**a**) Hematoxylin and eosin (**H**,**E**) staining (**b**) accompanied by statistical evaluation of the Organ Pathological Injury Score (scale bars: 100 μm and 20 μm). (**H**–**K**) Measurements of serum H_2_S content, NAMPT enzymatic activity, NAD^+^ levels, and ATP concentrations. Statistical significance: * *p* < 0.05, ** *p* < 0.01, *** *p* < 0.001 versus the specified control group; ns denotes no significance. Data are expressed as mean ± SD (n = 5 per group), analyzed using one-way ANOVA.

To model cerebral ischemia–reperfusion (I/R) injury, we utilized a MCAO/R protocol in rats. As shown in Figure 2A (Zea-Longa scoring), MCAO/R rats exhibited a marked threefold increase in neurological deficit scores compared to sham controls. Consistent with this, cerebral I/R injury also led to significant elevations in serum levels of LDH and NSE (1.6-fold and 1.4-fold, respectively; Figure 2B,C). Furthermore, cognitive function was severely impaired, as evidenced by prolonged escape latency and a reduced number of target platform crossings (Figure 2D). Cerebral infarct volume was notably increased by 38% relative to the sham group (Figure 2E). Histopathological examination revealed extensive necrosis in cortical tissue, a marked reduction in neuronal count, nuclear pyknosis, hyperchromasia or even nuclear lysis, cytoplasmic pallor, and abundant vacuole formation following I/R injury (Figure 2F,G).

Interestingly, treatment with NaHS (1.2, 2.4, or 4.8 mg/kg) dose-dependently ameliorated all of the above I/R-induced alterations, including the elevated neurological deficit score, increased serum LDH and NSE levels, cognitive dysfunction, neuronal loss, and histopathological injury score (Figure 2B–G). These findings indicate a robust protective effect of H_2_S against cerebral I/R injury in rats. In contrast, administration of FK866 (5 mg/kg) alone did not significantly affect any of these parameters. However, when combined with NaHS (4.8 mg/kg), FK866 effectively reversed the protective effects of NaHS on neurological deficits, serum LDH/NSE levels, learning and memory performance, neuronal count, and histopathological score (Figure 2B–G), suggesting that NAMPT mediates the neuroprotective action of H_2_S in this model.

Additionally, NaHS (1.2, 2.4, or 4.8 mg/kg) elevated serum H_2_S levels and significantly suppressed the I/R-induced decline in NAMPT activity, NAD^+^ content, and ATP levels. Notably, co-treatment with FK866 (5 mg/kg) markedly attenuated these effects of 4.8 mg/kg NaHS (Figure 2H–K), further supporting the involvement of the NAMPT/NAD^+^/ATP axis in the protective mechanism of H_2_S.

Collectively, these findings suggest that H_2_S mitigates cerebral I/R injury in rats by boosting ATP synthesis via the NAMPT–NAD^+^ signaling axis. These results align with the well-established concept that enhancing NAD^+^ bioavailability promotes ATP synthesis and protects against ischemic injury. However, they challenge the conventional view that H_2_S primarily exerts neuroprotection via anti-inflammatory or anti-oxidant mechanisms, instead highlighting a direct metabolic pathway through the NAMPT–NAD^+^ signaling axis.

### 2.3. Role of S-Sulfhydration Modification of NAMPT in NaHS Protecting Against H/R Injury in Rat Cerebrovascular ECs

**Figure 3:**
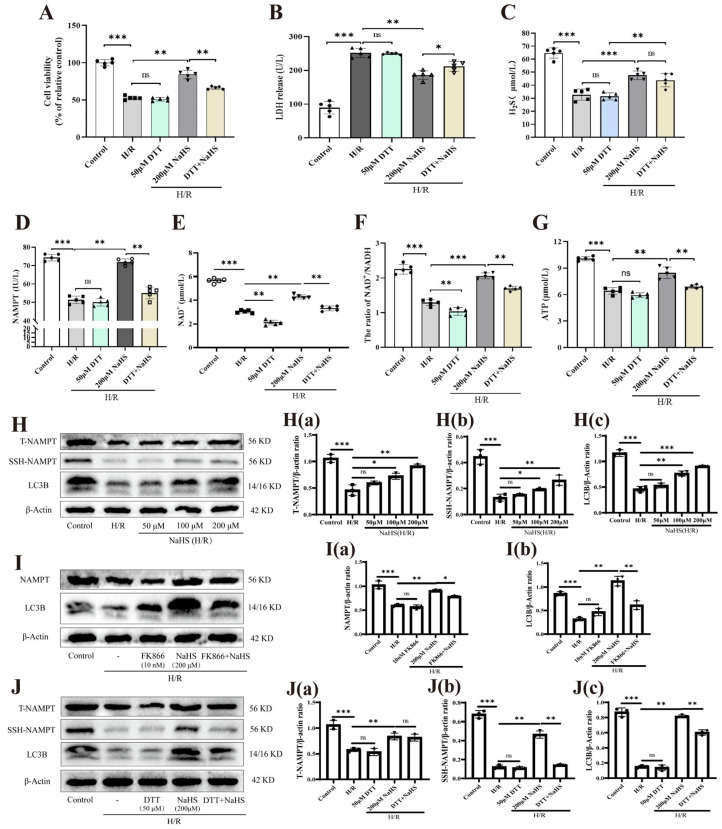
The effects of S-sulfhydration inhibitor on H_2_S-mediated alleviation of H/R injury in rat cerebrovascular ECs. (**A**–**G**) Cell viability, LDH release, H_2_S level, NAMPT activity, NAD^+^ level, the ratio of NAD^+^/NADH and ATP level in ECs (n = 5). (**H**,**H(a)**,**H(b)**,**H(c)**) Western blot analysis and quantification of total NAMPT (T-NAMPT), S-sulfhydration NAMPT (SSH-NAMPT) and LC3B after treatment with NaHS in ECs (n = 3). (**I**,**I(a)**,**I(b)**) Western blot analysis and quantification of T-NAMPT and LC3B after treatment with NaHS and/or FK866 in ECs (n = 3). (**J**,**J(a)**,**J(b)**,**J(c)**) Western blot analysis and quantification of T-NAMPT, SSH-NAMPT and LC3B after treatment with NaHS and/or DTT in ECs (n = 3). Statistical significance is denoted by * *p* < 0.05, ** *p* < 0.01, and *** *p* < 0.001 relative to specified control groups; “ns” indicates no significance. Values are expressed as mean ± SD; statistical comparisons employed one-way ANOVA.

As illustrated in Figure 3A–C, the S-sulfhydration blocker DTT (50 μM) did not alter baseline H_2_S levels, endothelial cell viability, or LDH release. Nevertheless, DTT significantly abrogated the inhibitory impacts of NaHS (200 μM) on these parameters. Further analysis shown in Figure 3D,E indicates that DTT (50 μM) not only potently suppressed the NaHS (200 μM)-driven upregulation of NAMPT activity in endothelial cells subjected to hypoxia/reoxygenation (H/R) stress but also prevented the associated rise in NAD^+^ content. Furthermore, DTT (50 μM) markedly reversed the ability of NaHS (200 μM) to attenuate the H/R-induced drop in the NAD^+^/NADH ratio and the depletion of ATP levels (Figure 3F,G). These observations support the conclusion that S-sulfhydration modification is integral to the protective mechanism of H_2_S against H/R injury in rat cerebrovascular endothelial cells.

Western blotting results (Figure 3H–J) revealed that NaHS (50, 100, and 200 μM) prevented the H/R-induced downregulation of NAMPT protein expression in rat cerebrovascular endothelial cells. This restorative effect of 200 μM NaHS was significantly diminished by FK866 (10 nM) (diminished by 1.2-fold, from 0.9039 to 0.7815, *p*-value = 0.0476). Additionally, blot analyses demonstrated that H/R injury reduced NAMPT S-sulfhydration by 5.6-fold (from 0.6767 to 0.1201, *p* < 0.001 compared to Control group). NaHS (50, 100, and 200 μM) robustly enhanced the expression of S-sulfhydrated NAMPT protein in endothelial cells following H/R insult, whereas DTT (50 μM) substantially weakened this promoting effect of 200 μM NaHS (reduced by 3.3-fold, from 0.4636 to 0.1402, *p*-value < 0.01). Taken together, these data indicate that post-translational S-sulfhydration of NAMPT plays a critical role in the protective effect of H_2_S against H/R injury in rat cerebrovascular ECs.

This finding is consistent with the emerging paradigm that protein S-sulfhydration mediates many of H_2_S’s cytoprotective effects, but it challenges the conventional view that NAMPT’s role in hypoxic/ischemic injury is regulated primarily at the transcriptional or phosphorylation level rather than through reversible thiol modification.

### 2.4. Improvement of NaHS on the Decreased Mitochondrial Function by H/R Injury in the ECs, and Effects of FK866 and DTT on the Improvement

**Figure 4:**
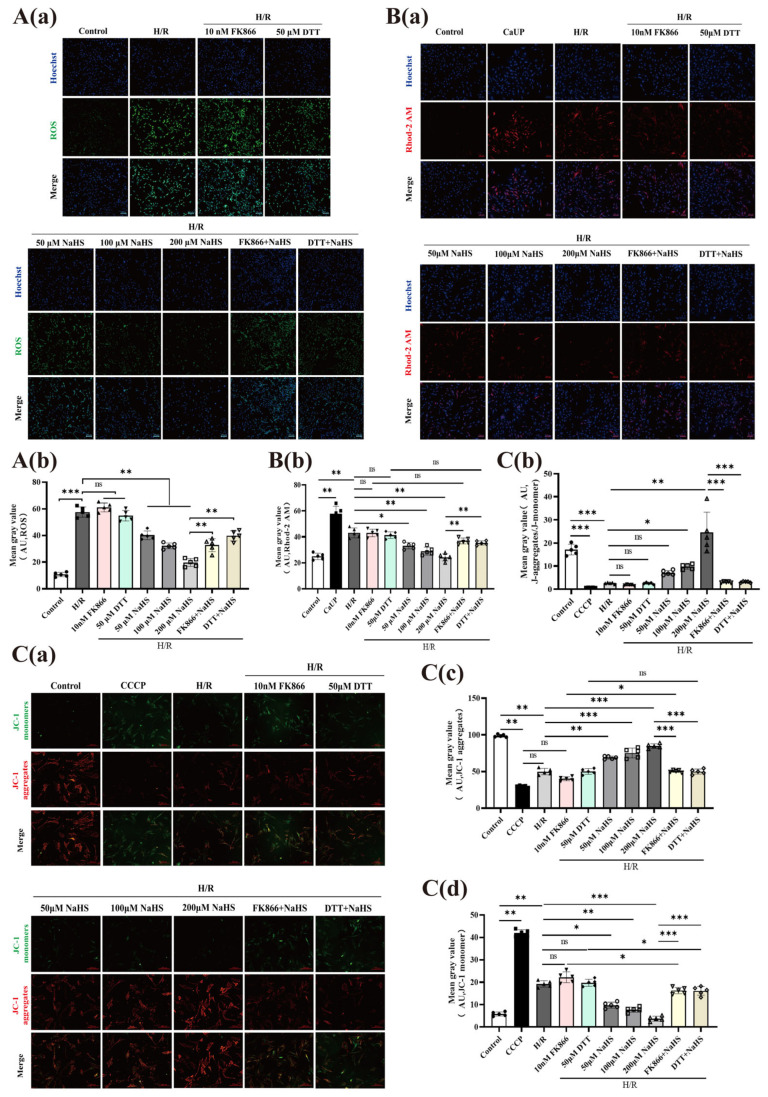
Effects of FK866 and DTT on H_2_S-mediated protection of mitochondrial function in rat cerebrovascular ECs subjected to H/R injury. (**A**) (**a**) Representative micrographs of ROS staining (green) alongside nuclear counterstaining with Hoechst (blue); corresponding quantitative bar graphs are provided. (**b**) Statistics of average fluorescence intensity of ROS in each group. Scale bar: 100 μm. (**B**) (**a**) Representative images of Rhod-2 AM-loaded ECs reflecting intracellular Ca^2+^ levels (red fluorescence intensity), (**b**) accompanied by statistical analysis of average fluorescence intensity of Ca^2+^ in each group. Scale bar: 100 μm. (**C**) Assessment of mitochondrial membrane potential using the JC-1 probe; bar graphs quantify the fluorescence intensities of JC-1 monomers (green) and aggregates (red); (**a**) Representative diagram of mitochondrial membrane potential staining labeled with JC-1 probe; (**b**) Statistics of average fluorescence intensity of JC-1 monomers (green); (**c**) Statistics of average fluorescence intensity of JC-1 aggregates (red); (**d**) Statistics of the fluorescence intensity ratio between JC-1 aggregates and JC-1 monomers. Scale bar: 200 μm. Data are presented as mean ± SD; statistical significance (* *p* < 0.05, ** *p* < 0.01, *** *p* < 0.001) versus indicated groups was determined by one-way ANOVA (n = 5); “ns” denotes no significance.

To evaluate intracellular ROS, calcium ion (Ca^2+^) dynamics, and mitochondrial membrane potential (ΔΨm), specific fluorescent probes were utilized. As demonstrated in Figure 4A,B, NaHS administration (50, 100, and 200 μM) markedly suppressed the H/R-triggered elevation of both ROS and Ca^2+^ levels in rat cerebrovascular ECs. Notably, this protective effect observed at 200 μM NaHS was significantly compromised by concurrent treatment with either FK866 (10 nM) or DTT (50 μM). Given that excessive intracellular ROS and Ca^2+^ accumulation can induce calcium overload—thereby destabilizing ΔΨm and impairing mitochondrial integrity—we further examined mitochondrial health via JC-1 staining. NaHS (50–200 μM) effectively prevented the H/R-mediated increase in JC-1 monomers (green fluorescence) and the concomitant loss of JC-1 aggregates (red fluorescence), signifying preservation of ΔΨm. However, this beneficial action of 200 μM NaHS was substantially reversed by FK866 (diminished by 7.9-fold, from 24.52 to 3.123, *p*-value < 0.001) or DTT (reduced by 7.5-fold, from 24.34 to 3.224, *p*-value < 0.001) (Figure 4C). Collectively, these findings indicate that H_2_S counteracts H/R-evoked mitochondrial deterioration in endothelial cells primarily through S-sulfhydration-dependent modification of NAMPT.

**Figure 5:**
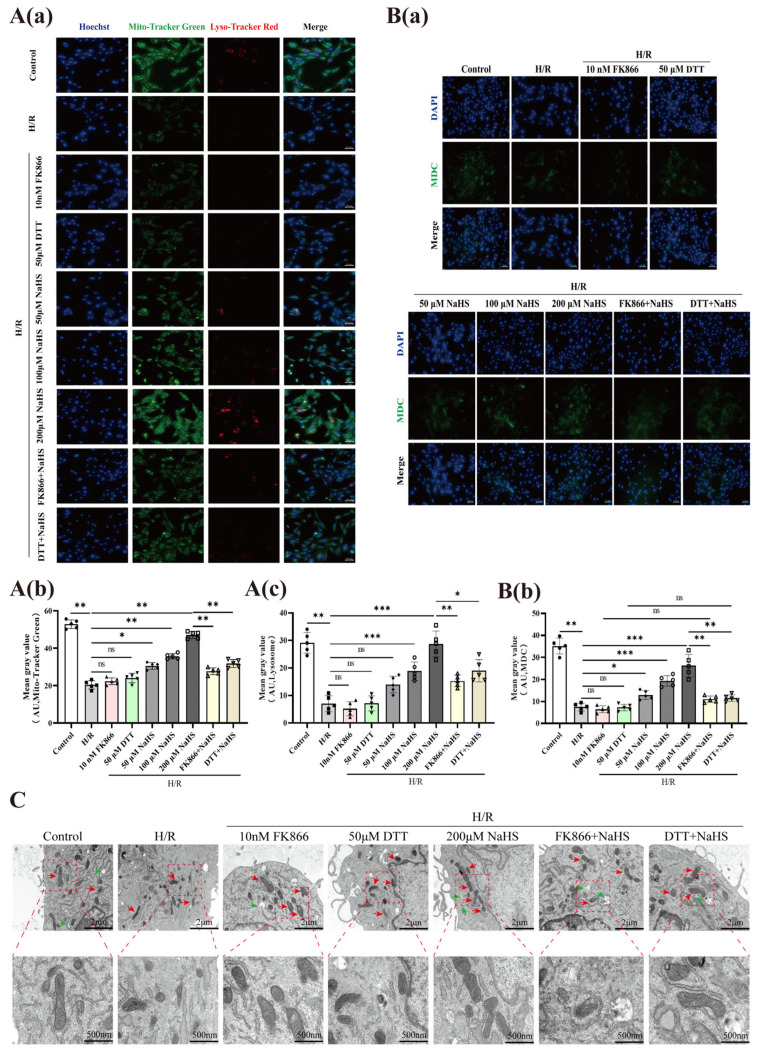
Effects of FK866 and DTT on H_2_S-induced autophagy in rat cerebrovascular ECs following H/R injury. (**A**) The ECs were stained by Mito-Tracker green and Lyso-Tracker red, and the statistical histograms show the mean gray value of the fluorescence; (**a**) Mito Tracker Green and Lyso Tracker Red probes represent the staining of mitochondria and lysosomes, respectively; (**b**) Statistics of mitochondrial average fluorescence intensity; (**c**) Statistics of average fluorescence intensity of lysosomes. Scale bar, 50 μm, n = 5. (**B**) MDC Staining of autophagosome and quantitative analysis of immunofluorescence intensity; (**a**) Representative staining image of MDC probe labeled autophagosome; (**b**) Statistics of average fluorescence intensity of autophagosomes. Scale bar, 50 μm, n = 5. (**C**) TEM images of the ultrastructure of mitochondria (the red arrow) and lysosome (the green arrow) in ECs. Scale bar, 2 μm and 500 nm, n = 3. Statistical significance is denoted by * *p* < 0.05, ** *p* < 0.01, and *** *p* < 0.001 versus the specified groups, with “ns” indicating no significance. Data are expressed as means ± SD (One-way ANOVA).

Organelles were visualized using the fluorescent probes Mito-Tracker Green, Lyso-Tracker Red, and MDC, respectively. As illustrated in Figure 5A,B, treatment with NaHS (50, 100, and 200 μM) significantly reversed the H/R-triggered decline in fluorescence intensity associated with mitochondria, lysosomes, and autophagosomes within rat cerebrovascular ECs. Notably, the augmenting influence of 200 μM NaHS was substantially abrogated by co-administration of FK866 (10 nM) or DTT (50 μM). Western blotting analysis of the autophagy-related protein LC3B revealed that NaHS (50, 100 and 200 μM) inhibited the H/R-induced down-regulation of LC3B protein expression in ECs, and this promoting effect of 200 μM NaHS was significantly attenuated by FK866 (10 nM) (diminished by 1.8-fold, from 1.126 to 0.6144, *p*-value < 0.01) or DTT (50 μM) (reduced by 1.4-fold, from 0.8149 to 0.6025, *p*-value < 0.01) (Figure 3H–J). In vivo experiments revealed that NaHS (1.2, 2.4, and 4.8 mg/kg) effectively prevented the I/R-induced suppression of LC3B expression in brain tissue, while the protective impact of the highest dose (4.8 mg/kg) was significantly overturned by FK866 (5 mg/kg) (reduced by 1.5-fold, from 1.035 to 0.6723, *p*-value < 0.01) or DTT (50 μg/kg) (diminished by 2.3-fold, from 0.9585 to 0.4186, *p*-value < 0.05) (Figure 7E–G). Immunohistochemical analysis (Figure 7H) further corroborated these findings, showing that 4.8 mg/kg NaHS robustly inhibited the I/R-driven loss of LC3B-positive cells in the brain, an effect that was considerably weakened by FK866 and DTT treatment. Furthermore, transmission electron microscopy (TEM) of endothelial ultrastructure indicated that 200 μM NaHS markedly alleviated H/R-induced mitochondrial injury and the depletion of autophagosomes in rat cerebrovascular ECs; however, FK866 (10 nM) and DTT (50 μM) distinctly nullified this cytoprotective action (Figure 5C). Collectively, these data indicate that H_2_S, via S-sulfhydration modification of NAMPT, promotes autophagic function after endothelial H/R injury, accelerates autophagy of damaged mitochondria, and thereby ameliorates endothelial cell damage.

Collectively, these data indicate that H_2_S, via S-sulfhydration modification of NAMPT, promotes autophagic function after endothelial H/R injury, accelerates autophagy of damaged mitochondria, and thereby ameliorates endothelial cell damage. This finding is consistent with the established protective role of H_2_S against ischemia–reperfusion injury and the pro-survival function of autophagy, but it challenges the conventional view that NAMPT primarily acts through NAD^+^ biosynthesis, instead revealing a novel post-translational mechanism—S-sulfhydration—that directly couples NAMPT to autophagic regulation.

### 2.5. Effect of DTT on the Protection of NaHS Against Cerebral I/R Injury in Rats

**Figure 6:**
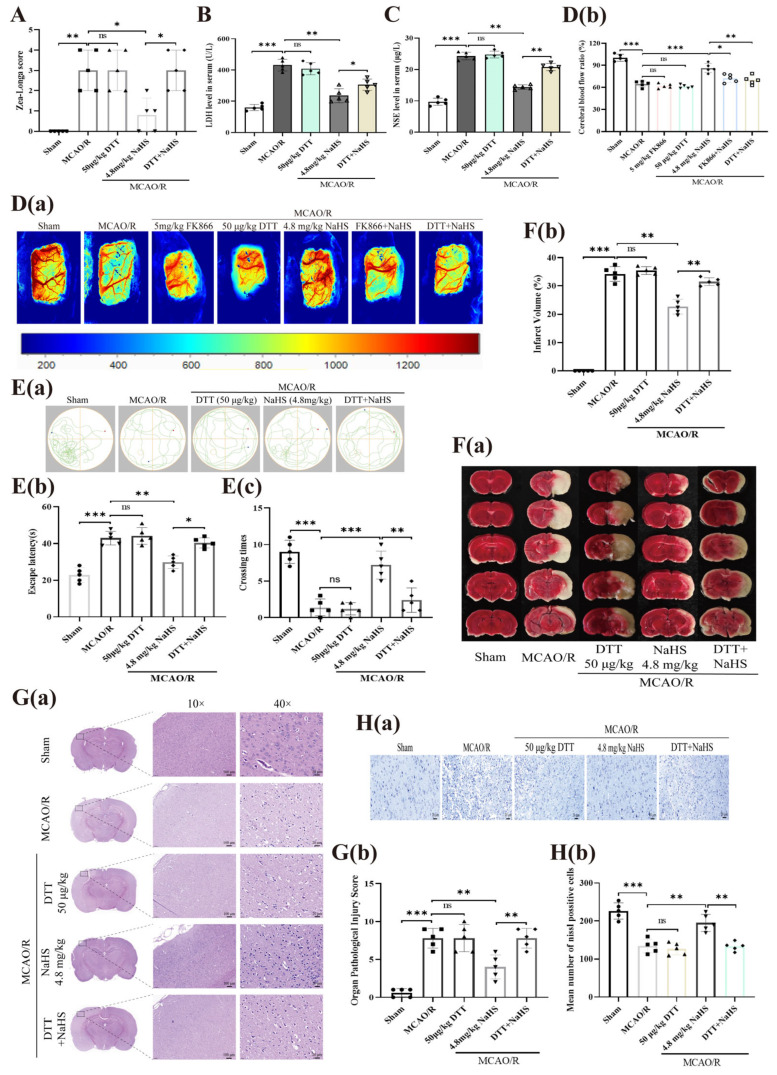
Effects of S-sulfhydration inhibitor on H_2_S-induced improvement of cerebral I/R injury in rats. (**A**) Assessment of neurological deficits via Zea-Longa scoring following MCAO/R. (**B**,**C**) LDH and NSE level in serum. (**D**) (**a**) Representative images of laser speckle contrast imaging for rat cerebral blood flow detection, (**b**) and the statistics of cerebral blood flow ratio. (**E**) Water maze test and the statistics of Escape latency and Crossing times; (**a**) Representative diagram of swimming trajectories of rats in each group; (**b**) Comparison of escape latency among different groups of rats; (**c**) Comparison of the number of times each group of rats crosses the platform. (**F**) (**a**) Representative TTC-stained brain sections and (**b**) quantitative analysis of infarct volume across groups. (**G**) (**a**) HE staining and (**b**) the statistics of Organ Pathological Injury Score. Scale bar, 100 μm and 20 μm. (**H**) (**a**) Nissl staining and (**b**) the statistics of Nissl positive cells. Scale bar, 20 μm. * *p* < 0.05, ** *p* < 0.01, *** *p* < 0.001 versus the specified control group; ns denotes no significance. Data are expressed as mean ± SD (n = 5 per group), analyzed using one-way ANOVA.

As presented in Figure 6A, the S-sulfhydration inhibitor DTT (50 μg/kg) significantly counteracted the reduction in neurological deficit scores induced by NaHS (4.8 mg/kg) following cerebral I/R injury in rats. Moreover, DTT (50 μg/kg) markedly attenuated the suppressive effects of NaHS (4.8 mg/kg) on serum LDH and NSE levels post-I/R insult (Figure 6B,C). These observations imply that S-sulfhydration modification plays a critical role in the neuroprotective mechanism of H_2_S against cerebral I/R injury.

Additionally, Figure 6D demonstrates that while NaHS (4.8 mg/kg) (increased by 1.3-fold, from 64.58% to 86.3%, *p*-value < 0.001) significantly accelerated the recovery of cerebral blood flow after I/R injury, this beneficial effect was substantially inhibited by both FK866 (5 mg/kg) (reduced by 1.2-fold, from 86.3% to 72.34%, *p*-value < 0.05) and DTT (50 μg/kg) (reduced by 1.2-fold, from 86.3% to 69.93%, *p*-value < 0.01). Data obtained from the Morris water maze assay indicated that DTT (50 μg/kg) significantly abolished the therapeutic benefits of NaHS (4.8 mg/kg) regarding learning and memory deficits triggered by I/R insult (Figure 6E). Moreover, administration of DTT (50 μg/kg) counteracted the NaHS (4.8 mg/kg)-driven shrinkage in cerebral infarct volume, restoration of neuronal populations, and alleviation of histopathological damage scores (Figure 6F–H). These observations offer additional support for the critical involvement of S-sulfhydration in the neuroprotective mechanisms of H_2_S against cerebral I/R injury in rats.

**Figure 7:**
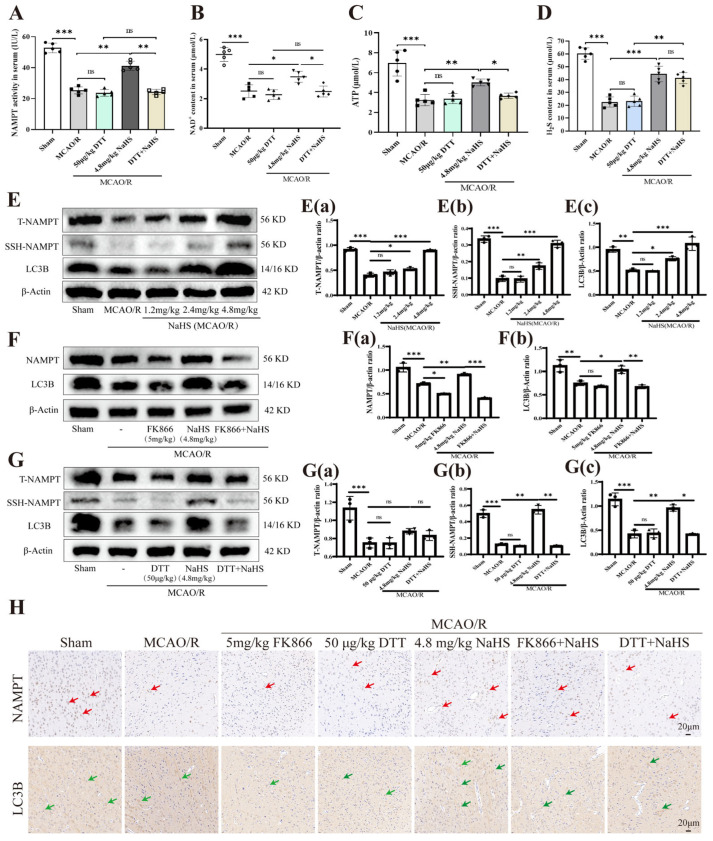
Effects of FK866 and DTT on protein expression in rat brain tissue. (**A**–**D**) NAMPT activity, NAD^+^ content ATP level and H_2_S content in serum, n = 5. (**E**,**E**(**a**),**E**(**b**),**E**(**c**)) Western blot analysis and quantification of total NAMPT (T-NAMPT), S-sulfhydration NAMPT (SSH-NAMPT) and LC3B after treatment with NaHS in rat brain tissue, n = 3. (**F**,**F**(**a**),**F**(**b**)) Western blot analysis and quantification of T-NAMPT and LC3B after treatment with NaHS and/or FK866 in rat brain tissue, n = 3. (**G**,**G**(**a**),**G**(**b**),**G**(**c**)) Western blot analysis and quantification of T-NAMPT, SSH-NAMPT and LC3B after treatment with NaHS and/or DTT in rat brain tissue, n = 3. (**H**) Immunohistochemical staining was used to visualize the positive expression of NAMPT and LC3B proteins in the cortical region of the experimental group. Results are presented as the number of cells showing positive expression for NAMPT and LC3B. Positive immunoreactivity, characterized by a brownish-yellow hue, is highlighted by red arrows (NAMPT) and green arrows (LC3B). Scale bar, 20 μm, n = 5. * *p* < 0.05, ** *p* < 0.01,*** *p* < 0.001 compared to the specified control groups, ns indicates no significance. All data are presented as mean ± SD, derived from one-way ANOVA.

Figure 7A through Figure 7D illustrate that DTT (50 μg/kg) exerted no influence on circulating H_2_S concentrations following I/R trauma. Nevertheless, by suppressing serum NAMPT enzymatic activity, DTT mitigated the NaHS (4.8 mg/kg)-provoked elevations in serum NAD^+^ and ATP levels. Such results imply that S-sulfhydration modification is integral to the H_2_S-facilitated boost in ATP synthesis mediated via the NAMPT–NAD^+^ pathway.

Western blotting analyses (Figure 7E–G) revealed that NaHS treatment at doses of 1.2, 2.4, and 4.8 mg/kg markedly prevented the I/R-associated decline in NAMPT protein abundance within brain tissue. The enhancing impact observed with 4.8 mg/kg NaHS was substantially diminished by co-administration of FK866 (5 mg/kg) (diminished by 2.2-fold, from 0.9082 to 0.4066, *p*-value < 0.001). Blot data further demonstrated that I/R injury reduced NAMPT S-sulfhydration by 4-fold (from 0.4974 to 0.1243, *p* < 0.001 compared to Sham group). NaHS (1.2, 2.4, and 4.8 mg/kg) robustly upregulated the expression of S-sulfhydrated NAMPT in the brain post-I/R, an effect that was profoundly reversed by DTT (50 μg/kg) (reduced by 5.3-fold, from 0.5462 to 0.1023, *p*-value < 0.01) at the 4.8 mg/kg NaHS dose. Immunohistochemical findings (Figure 7H) additionally confirmed that NaHS (4.8 mg/kg) effectively restrained the I/R-induced loss of NAMPT-positive cells in brain parenchyma, a protective outcome that was notably compromised by both FK866 (5 mg/kg) and DTT (50 μg/kg). Taken together, these findings establish that S-sulfhydration of NAMPT constitutes a key mechanism underlying the protective efficacy of H_2_S against cerebral I/R injury.

These findings are consistent with the well-established protective role of H_2_S against cerebral I/R injury, but they challenge the conventional view that NAMPT primarily exerts its effects through its enzymatic activity (e.g., NAD^+^ biosynthesis) without considering post-translational modifications such as S-sulfhydration.

### 2.6. Expression and Purification of His-NAMPT and Analysis of NaHS-Induced S-Sulfhydration at Cys401, Cys397 and Cys39 by LC-MS/MS In Vitro

**Figure 8:**
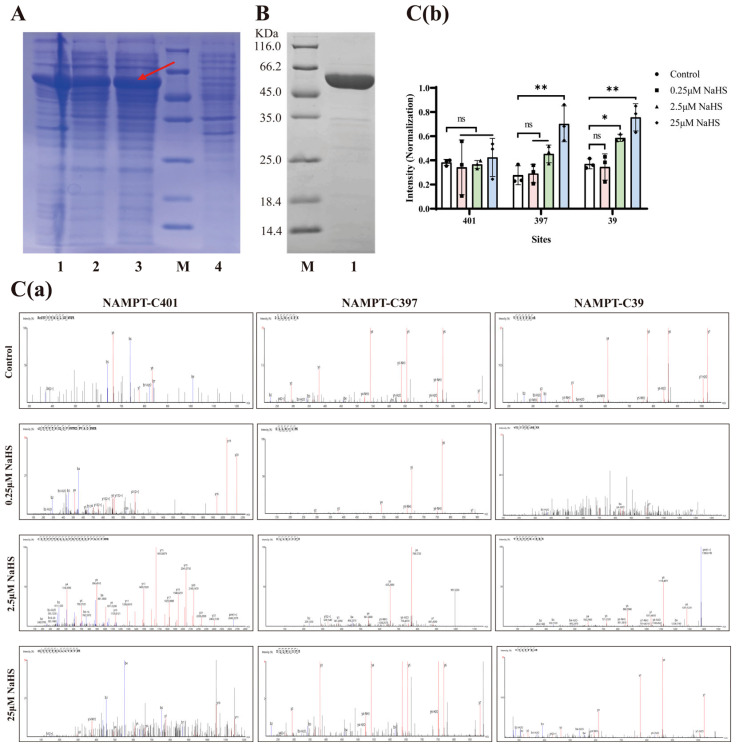
Expression and purification of His-NAMPT, and analysis of NaHS-induced S-sulfhydration at Cys401, Cys397 and Cys39 by LC-MS/MS in vitro. (**A**) Coomassie Brilliant Blue staining assessing recombinant His-NAMPT expression. 1: Induced expression of pelleted protein after lysis; 2: Induced expression of supernatant protein after lysis; 3: Total protein in lysate following induced expression and disruption; M: Marker, Protein molecular weight standard (116.0/66.2/45.0/35.0/25.0/18.4/14.4 kDa); 4: Total protein from lysate after uninduced expression and lysis. The red arrow indicates the recombinant protein. (**B**) SDS-PAGE electropherogram for purified His-NAMPT protein. M: Marker; 1: Purified supernatant protein. (**C**) S-sulfhydration at Cys401, Cys397, and Cys39; (**a**) Mass spectrometry analysis of the modification of Cys401, Cys397, and Cys39 sites of NAMPT by S-sulfhydration induced by different concentrations of NaHS; (**b**) Statistical analysis of relative quantification of ion intensity for Cys401, Cys397, and Cys39 sites by mass spectrometry. Mass spectrometry analysis images indicated: NAMPT-Cys401 400-415: KCSYVVTNGLGVNVFK in the control group; NAMPT-Cys401 401-424: CSYVVTNGLGVNVFKDPVADPNKR in the 0.25 μM NaHS group and 2.5μM NaHS group; NAMPT-Cys401 401-415: CSYVVTNGLGVNVFK in the 25 μM NaHS group; NAMPT-Cys397 393-400: DLLNCSFK in the control group, 0.25 μM NaHS group, 2.5 μM NaHS group and 25 μM NaHS group; NAMPT-Cys39 33-40: VYSYFECR in the control group and 25 μM NaHS group; NAMPT-Cys39 33-43: VYSYFECREKK in the 0.25 μM NaHS group; NAMPT-Cys39 33-42: VYSYFECREK in the 2.5 μM NaHS group, and the relative quantification of S-sulfhydration at Cys401, Cys397, and Cys39 sites (with ion intensity normalization). Statistical significance is denoted by * *p* < 0.05 and ** *p* < 0.01 versus specified groups; “ns” indicated no significance. Data are presented as mean ± SD (n = 3; one-way ANOVA).

To pinpoint specific S-sulfhydration sites on NAMPT induced by H_2_S exposure, we constructed a prokaryotic expression vector, His-NAMPT-pET28a(+), and transformed it into Escherichia coli for the production of His-tagged recombinant NAMPT. As illustrated in Figure 8A, small-scale culture of a positive clone followed by SDS-PAGE analysis revealed a distinct band (marked by a red arrow), confirming successful gene expression upon IPTG induction. Subsequent large-scale cultivation and ultrasonic lysis yielded a His-tagged recombinant protein (~4 kDa tag). SDS-PAGE profiling indicated an apparent molecular weight of approximately 59 kDa for the purified product, aligning precisely with the theoretical mass of the target protein. The soluble fraction from large-scale cultures underwent purification and concentration via Ni-NTA affinity chromatography. The efficacy of this purification process was further validated by SDS-PAGE analysis, as shown in Figure 8B.

To identify potential S-sulfhydration modifications on the recombinant His-NAMPT, we performed liquid chromatography-tandem mass spectrometry (LC-MS/MS). As depicted in Figure 8C(a), baseline S-sulfhydration was detected at residues Cys401, Cys397, and Cys39 even in the absence of NaHS treatment. To assess the modulatory impact of H_2_S on these sites, the recombinant protein was exposed to varying concentrations of NaHS (0.25, 2.5, and 25 μM). Quantitative analysis of normalized ion intensities across treatment groups (Figure 8C(b)) demonstrated that NaHS administration did not alter S-sulfhydration levels at Cys401. Conversely, higher concentrations of NaHS (2.5 and 25 μM) significantly augmented S-sulfhydration at both Cys397 and Cys39, whereas the lowest concentration (0.25 μM) exerted no discernible effect on these two residues. Collectively, these findings indicate that H_2_S specifically enhances the S-sulfhydration of NAMPT at Cys397 and Cys39.

## 3. Discussion

The present study firstly demonstrates that H_2_S protects against cerebral I/R injury by sulfhydrating NAMPT, thereby activating the NAD^+^-ATP axis and enhancing autophagic activity, which collectively safeguard cerebrovascular ECs and brain tissue. These findings provide new insights into the mechanisms of H_2_S as a neuroprotective molecule and establish a theoretical foundation for the development of therapeutic strategies against cerebral I/R injury.

Cerebral I/R injury is a major pathological contributor to neurological deficits following ischemic stroke, involving mechanisms such as oxidative stress, calcium overload, inflammatory responses, and mitochondrial dysfunction [22,23,24]. Although current recanalization therapies (e.g., thrombolysis and thrombectomy) can partially restore blood flow, they are often accompanied by reperfusion injury, which limits their clinical benefits [25,26]. Thus, identifying adjunctive strategies to alleviate I/R injury is of significant clinical importance. In recent years, the gas signaling molecule H_2_S has attracted increasing attention due to its anti-inflammatory, antioxidant, and anti-apoptotic effects observed across multiple disease models [27,28,29]. This study further narrows its mechanistic focus to NAMPT—a central regulatory enzyme in energy metabolism—and its post-translational modification.

NAMPT serves as the rate-limiting enzyme in the NAD^+^ salvage pathway and plays a key role in maintaining cellular energy homeostasis and mitochondrial function [30,31,32]. Our results indicate that, in a H/R model, H_2_S enhances NAMPT activity via S-sulfhydration, thereby promoting NAD^+^ biosynthesis, increasing ATP production, improving mitochondrial membrane potential, and reducing ROS accumulation. This mechanism is particularly prominent in primary cerebrovascular ECs, suggesting that the H_2_S-NAMPT-NAD^+^ axis plays a central role in cerebral I/R injury protection.

Beyond regulating energy metabolism, this study also revealed that H_2_S promotes autophagic activity, accelerating autophagy of damaged mitochondria and further enhancing cellular homeostasis. Autophagy, an adaptive response under stress, exhibits a dual role in I/R: moderate activation clears damaged organelles, whereas excessive activation may trigger cell death [33]. The H_2_S-mediated enhancement of autophagy observed here is clearly protective, a process potentially associated with SIRT1 activation—which is NAD^+^-dependent—thus providing new clues to the crosstalk between energy metabolism and autophagy. Although our study demonstrated that H_2_S promotes autophagic activity in injured cells—evidenced by LC3B expression, fluorescence detection of autophagosomes and lysosomes, and TEM-based observation of their numerical changes at the ultrastructural level—we acknowledge the lack of definitive flux experiments to corroborate this conclusion, which represents a limitation of our experimental design.

From a molecular perspective, protein S-sulfhydration is a reversible post-translational modification involving the addition of a sulfhydryl group to cysteine residues, thereby modulating protein function, subcellular localization, and interactions [34,35]. This study experimentally demonstrates that H_2_S directly modifies key cysteine residues in NAMPT, enhancing its enzymatic activity and stability. Furthermore, using LC-MS/MS technology [36], we detected that H_2_S significantly promotes the S-sulfhydration of Cys39 and Cys397 sites in NAMPT under in vitro conditions. This finding not only deepens our understanding of H_2_S signaling but also expands the known roles of redox modifications in cerebrovascular diseases.

Our present study demonstrates that H_2_S protects against cerebral I/R injury by S-sulfhydrating NAMPT, thereby enhancing NAD^+^ biosynthesis, mitochondrial function, and autophagy in cerebrovascular ECs. Partial validation results are similar to several recent reports showing that exogenous H_2_S promotes PINK1/Parkin-mediated mitophagy to alleviate cerebral I/R injury [37]. Moreover, the role of NAMPT as an upstream regulator of autophagy is supported by evidence that astrocyte-derived exosomal NAMPT activates AMPK/mTOR signaling to induce neuroprotection [38]. However, notable differences exist. While Zhao et al. [38] focused on NAMPT derived from astrocytic exosomes acting on neurons, our study identifies endothelial intracellular NAMPT as the direct target of H_2_S-mediated S-sulfhydration. Additionally, a study on spinal cord I/R reported that a slow-releasing H_2_S donor (GYY4137) inhibited neuronal cell death via anti-PANoptosis, rather than mitophagy [39], suggesting context-dependent mechanisms across different tissues. A cardiac study further showed that H_2_S-dependent S-sulfhydration of mitochondrial complexes I–V controlled respiration in diabetic hearts [40], reinforcing the importance of S-sulfhydration as a conserved regulatory mechanism across organ systems. Together, these convergent and divergent lines of evidence highlight that the H_2_S-NAMPT-NAD^+^ axis operates in a cell-type- and modification-specific manner, offering a refined therapeutic target for next-generation neuroprotective strategies.

Nevertheless, certain constraints warrant acknowledgment. Primarily, as all investigations were confined to cellular and animal models, the absence of validation using clinical specimens from stroke patients restricts the immediate translational applicability of these results. Additionally, the modest sample size may compromise the statistical robustness of specific findings; consequently, future inquiries employing larger cohorts and multi-center designs are essential. Moreover, given that this research predominantly examined endothelial cells (ECs), the influence of H_2_S on other neural constituents, including neurons and glial cells, remains to be clarified. Furthermore, while FK866 and DTT are widely used tools to inhibit NAMPT and reduce disulfide bonds/S-sulfhydration, respectively, we acknowledge their potential for off-target effects. Finally, systematic assessments regarding the pharmacokinetic profiles of H_2_S donors, optimal therapeutic timeframes, and long-term safety parameters are required.

Furthermore, although we detected that H_2_S significantly induces S-sulfhydration at Cys39 and Cys397 of NAMPT in vitro using LC-MS/MS at the designed endpoint of our experiments, we have not yet performed functional validation for these specific sites. Therefore, we can only conclude that H_2_S exerts a protective effect against hypoxic injury both in vitro and in vivo through S-sulfhydration of NAMPT, rather than stating that H_2_S acts specifically via S-sulfhydration of NAMPT at Cys39 and Cys397. We explicitly state this as a limitation and suggest future experiments.

Despite these limitations, the findings of this study hold substantial scientific and clinical significance. We propose a novel signaling pathway wherein H_2_S S-sulfhydrates NAMPT, upregulates NAD^+^ levels, improves mitochondrial function, and activates protective autophagy, thereby alleviating I/R-induced cerebrovascular endothelial injury. This mechanism not only identifies a potential therapeutic target for ischemic stroke but also provides a rationale for developing H_2_S-based neuroprotective agents. Future research should focus on: (1) validating this pathway in more human-relevant models such as organoids or humanized systems; (2) developing small-molecule regulators targeting NAMPT S-sulfhydration; and (3) investigating the efficacy of H_2_S in comorbid models such as diabetic stroke.

## 4. Materials and Methods

### 4.1. Regents and Drugs

Sodium hydrosulfide (NaHS) was purchased from Sigma (St. Louis, MO, USA, Catalog #: 161527). Daporinad (FK866), dithiothreitol (DTT), and Cell Counting Kit-8 (CCK-8) were purchased from MedChemExpress (Beijing, China, Catalog #: HY-50876, HY-15917, HY-K0301). Polyclonal antibodies against Factor VIII (AF0156) and β-Actin (AF7018) were obtained from Affinity Biosciences. The NAMPT monoclonal antibody was purchased from Proteintech (Wuhan, China, Catalog #: 66385-1-Ig). The LC3B antibody and secondary antibody were purchased from Servicebio (Wuhan, China, Catalog #: GB113801, GB23303). The hydrogen sulfide (H_2_S) content assay kit was purchased from Elabscience (Shanghai, China, Catalog #: E-BC-K355-M). The lactate dehydrogenase (LDH) assay kit was purchased from Nanjing Jiancheng Bioengineering Institute (Nanjing, China, Catalog #: A020-2-2). The adenosine triphosphate (ATP) assay kit was purchased from Jingmei Biotechnology (Yancheng, China, Catalog #: JM-10331R2). The nicotinamide phosphoribosyltransferase (NAMPT), nicotinamide adenine dinucleotide (NAD^+^) and Neuronal specific enolase (NSE) assay kits were purchased from Jiangsu Meimian Industrial Co., Ltd. (Yancheng China, Lot #: MM-0953R2, MM-50259R2, MM-0069R2). Assay kits for reactive oxygen species (ROS), calcium ion (Ca^2+^), mitochondrial membrane potential, mitochondria, lysosomes, and autophagosomes were purchased from Beyotime (Shanghai, China, Catalog #: S0033S, S1062, C2006, C1996, C1046, C3018). MCAO filament (L3600, Jialing Biotech, Guangzhou, China).

### 4.2. Experimental Animals

Sprague-Dawley (SD) rats, aged 4–6 weeks with body weights ranging from 200 to 220 g, were procured from the Experimental Animal Center at Anhui Medical University. Throughout the study, animals were accommodated within the university’s Animal Center facility, enjoying unrestricted access to both diet and hydration. Environmental parameters were strictly regulated, maintaining relative humidity at 54 ± 2% and ambient temperature at 22 ± 2 °C. Every experimental maneuver received formal approval from the Ethics Committee of Anhui Medical University (Certification No. LLSC 20200829; Certification date: 1 March 2020) and was executed in full compliance with protocols set forth by the institution’s Animal Care and Use Committee. Furthermore, these procedures adhered to the ethical standards detailed in the National Institutes of Health (NIH) Guide for the Care and Use of Laboratory Animals (Publication No. 85-23, revised 2011).

Randomization and Blinding: Rats were randomly assigned to experimental groups (e.g., sham, model, and treatment) using a computer-generated randomization sequence. The allocation sequence was concealed in opaque sealed envelopes and was performed by an investigator not involved in any subsequent procedures. All surgical procedures, including induction of ischemia and drug administration, were carried out by a researcher blinded to group assignment. Neurological deficit scores were assessed by an experienced observer who was unaware of the treatment conditions. For infarct volume analysis and histological evaluation, brain sections were coded, and measurements were performed by a technician blinded to the experimental groups. The blinding was maintained until all data collection and primary analyses were completed.

### 4.3. Primary Cultures of Rat Cerebrovascular ECs and Identification

The isolation and subsequent cultivation of cerebrovascular endothelial cells (ECs) followed established methodologies [41]. Briefly, SD rats were anesthetized prior to brain extraction; harvested tissues were immediately immersed in ice-cold phosphate-buffered saline (PBS) containing penicillin and streptomycin. Under microscopic visualization, microvessels, specifically including the basilar and middle cerebral arteries, were meticulously dissected. These vascular segments were minced and subjected to enzymatic digestion using collagenase within a 37 °C water bath, accompanied by periodic gentle agitation. Post-digestion, the suspension underwent centrifugation, after which the supernatant was aspirated. The remaining pellet was resuspended in specialized endothelial growth medium and plated into culture flasks. Incubation occurred at 37 °C in an atmosphere enriched with 5% CO_2_. Twenty-four hours post-seeding, the medium was exchanged to eliminate non-adherent debris. Cultures were monitored routinely, with media replacements performed at regular intervals. Upon reaching approximately 80% confluency, cells were subcultured. Phenotypic identification was confirmed via immunofluorescence staining targeting Factor VIII, a specific marker for endothelial cells.

### 4.4. Establishment of H/R Injury

The H/R injury model in ECs was constructed according to previously reported protocols [42]. In summary, cells underwent washing with PBS, followed by replacement of the standard medium with glucose-free formulation. Subsequently, ECs were exposed to a hypoxic environment comprising 1% O_2_, 95% N_2_, and 4% CO_2_ for a duration of 4 h. Immediately following hypoxic insult, the medium was swapped for complete endothelial cell medium (ECM), and cultures were returned to normoxic conditions (37 °C, 95% air, 5% CO_2_) to facilitate a 6 h reoxygenation period. Control groups remained under continuous normoxic conditions without hypoxic exposure.

Regarding experimental grouping and pharmacological intervention, subjects were allocated into the following cohorts: Control, H/R, NaHS (50, 100, or 200 μM), FK866 (10 nM), DTT (50 μM), FK866 (10 nM) combined with NaHS (200 μM), and DTT (50 μM) combined with NaHS (200 μM). With the exception of the Control group, all other cohorts underwent H/R induction followed by a 24 h drug treatment regimen. NaHS, FK866, or DTT was added to the culture medium 1 h after H/R. Stock solutions of NaHS, FK866, and DTT were prepared in physiological saline and utilized immediately upon preparation to ensure stability.

### 4.5. Determination of Cell Viability and Biochemical Measurement

Sample Preparation Protocol: Cell Samples: After subjecting the cells to H/R modeling and treatment, first collect the cell culture supernatant into centrifuge tubes. Subsequently, detach the cells and collect them into new centrifuge tubes. Resuspend the cell pellet using the previously collected supernatant. The resulting cell suspension is then subjected to low-temperature ultrasonication for lysis. Post-lysis, samples were centrifuged at 4 °C and 12,000× *g* for 10 min. The resulting supernatant was collected and transferred to a fresh centrifuge tube for low-temperature storage.

Serum Samples Preparation: Following the conclusion of the experiment, anesthetize the rats and open the abdominal cavity to expose the heart. Prior to cardiac perfusion, draw an appropriate amount of blood using a syringe. The blood samples were allowed to clot at room temperature for 10 min, followed by centrifugation at 4 °C and 3000× *g* for 10 min. Collect the supernatant for subsequent analysis

Cell viability was quantified via the CCK-8 assay by measuring absorbance at 450 nm. Concentrations of lactate dehydrogenase (LDH), neuron-specific enolase (NSE), hydrogen sulfide (H_2_S), nicotinamide phosphoribosyltransferase (NAMPT) activity, nicotinamide adenine dinucleotide (NAD^+^), and adenosine triphosphate (ATP) were determined using commercially available kits strictly following the manufacturers’ instructions.

### 4.6. Western Blotting

Protein extraction and Western blot analysis were conducted as follows: Cells were washed twice with ice-cold phosphate-buffered saline (PBS) and lysed using RIPA lysis buffer. Protein concentration was assessed via the bicinchoninic acid (BCA) method. Equal amounts of protein were mixed with loading buffer, denatured by boiling, and separated by sodium dodecyl sulfate–polyacrylamide gel electrophoresis (SDS-PAGE). Proteins were then electrotransferred onto polyvinylidene difluoride (PVDF) membranes. Membranes were blocked with Tris-buffered saline containing 0.1% Tween-20 (TBST) supplemented with 5% non-fat skim milk for 2 h at room temperature, followed by overnight incubation with primary antibodies at 4 °C. After thorough washing, membranes were incubated with horseradish peroxidase (HRP)-conjugated secondary antibodies for 2 h at room temperature. Immunoreactive bands were visualized using enhanced chemiluminescence (ECL) and captured with a digital imaging system. Densitometric analysis of target bands was performed using ImageJ software (Rasband, W.S., ImageJ, U. S. National Institutes of Health, https://imagej.net/ij/ URL (accessed on 4 November 2025)).

### 4.7. Biotin Switch Assay

The biotin switch technique was employed to detect S-sulfhydrated proteins in endothelial cells (ECs) and brain tissues, adapted from a previously established protocol with minor modifications [43]. Briefly, protein lysates were treated with methyl methanethiosulfonate (MMTS) at a final concentration of 25 mmol/L and incubated at 50 °C for 20 min under constant agitation in a water or metal bath. Subsequently, two volumes of acetone were added, mixed by gentle inversion, and proteins were precipitated at −20 °C for 20 min. Following centrifugation at 15,000× *g* for 10 min at 4 °C, the supernatant was discarded. The pellet was resuspended in 1 mL of acetone and re-centrifuged under identical conditions. After removing the supernatant, an additional dry centrifugation step was carried out to eliminate residual liquid (this wash may be omitted if sample quantity is limited). The resulting pellet was resuspended in 50–400 μL of HEN buffer containing 4 mmol/L Biotin-HPDP (prepared as a 40 mmol/L stock in dimethyl sulfoxide) and incubated at 37 °C for 2 h. After another centrifugation at 15,000× *g* for 10 min at 4 °C, the supernatant was transferred to a new 1.5 mL tube as the final sample. An equal volume of non-reducing SDS loading buffer (5% SDS, 250 mmol/L Tris-HCl pH 6.8, 0.01% bromophenol blue) was added, thoroughly mixed, and incubated at 37 °C for 5 min. Samples were then resolved by SDS-PAGE and subjected to Western blot analysis.

### 4.8. Fluorescence Staining Assay

Fluorescent staining was performed according to the manufacturer’s instructions (Beyotime, China). Mitochondrial Membrane Potential (JC-1): “Cells were incubated with 2 µM JC-1 at 37 °C for 20 min. CCCP (10 µM, 30 min) was used as a positive control for depolarization.” Mitochondrial Ca^2+^ (Rhod-2 AM): “Cells were loaded with 5 µM Rhod-2 AM for 20 min at 37 °C, washed, and imaged. CaUp (2 µM, 30 min) was used as a positive control for depolarization.” Mitochondrial ROS (DCFH-DA): “Cells were loaded with 10 mM DCFH-DA for 20 min at 37 °C.” Autophagy (mitochondrion, autophagosome and lysosome): “Cells were incubated with 100 nM Mito-Tracker Green, 50 µM MDC or 100 nM Lyso-Tracker Red at 37 °C for 30 min.” Following the incubation period, cells underwent rinsing with phosphate-buffered saline (PBS) and were promptly examined under a fluorescence microscope within a light-shielded environment. Fluorescent signals were subsequently recorded and quantified by calculating the mean fluorescence intensity via ImageJ software (Rasband, W.S., ImageJ, U. S. National Institutes of Health, https://imagej.net/ij/).

### 4.9. Transmission Electron Microscopy

Briefly, cells from different treatment groups were collected with TEM fixative, processed according to standard transmission electron microscopy (TEM) sample preparation procedures, and observed under a TEM (HITACHI, Tokyo, Japan). Images were acquired and analyzed.

### 4.10. MCAO/R Model

The ischemia/reperfusion (I/R) model was induced in rats through middle cerebral artery occlusion/reperfusion (MCAO/R) [44]. Specifically, Sprague-Dawley rats (220–250 g) were subjected to a 12 h fasting regimen prior to surgical intervention and anesthetized via intraperitoneal administration of sodium pentobarbital (40 mg/kg). Following a midline cervical incision, the right common carotid artery (CCA) was isolated, and a suture was positioned for subsequent use. The proximal CCA was temporarily occluded with an arterial clip, while the external carotid artery was ligated. A minor incision was created on the CCA between the clip and the suture site. A nylon monofilament with a rounded tip (~220 μm diameter) was introduced into the CCA and advanced to the MCA origin to obstruct blood flow. Reperfusion was initiated by withdrawing the suture after 1.5 h. Sham-operated controls underwent identical surgical procedures excluding suture insertion. Core body temperature was strictly maintained at 37.0 ± 0.5 °C throughout the perioperative period. Post-anesthesia neurological function was evaluated using a standardized scoring scale; a score of ≥2 indicated successful model induction.

Neurological Deficit Score Evaluation: The Zea-Longa score quantifies the severity of neurological deficits in rats by observing their spontaneous behaviors in the awake state. Scores range from 0 to 4, with higher scores indicating more severe neurological impairment. References to the Supplementary Table S1 are provided for the detailed criteria. The text explicitly states that all behavioral and histopathological assessments were performed by investigators blinded to the experimental groups.

Regarding animal grouping and therapeutic administration, subjects were allocated into the following cohorts: Sham, MCAO/R, NaHS (1.2, 2.4, 4.8 mg/kg), FK866 (5 mg/kg), DTT (50 μg/kg), FK866 (5 mg/kg) + NaHS (4.8 mg/kg), and DTT (50 μg/kg) + NaHS (4.8 mg/kg). Except for the Sham group, all other groups underwent MCAO/R modeling and were subsequently treated with drugs for 7 days. NaHS, FK866, and DTT were prepared using physiological saline and were formulated freshly before use. NaHS or FK866 was administered via intraperitoneal injection 30 min before MCAO and once daily for the subsequent 6 days. DTT was administered via stereotaxic intracerebroventricular injection 30 min before MCAO and once daily for the subsequent 6 days.

### 4.11. Morris Water Maze Assay

Spatial learning and memory capabilities were assessed using the Morris water maze paradigm. The experimental setup comprised a circular pool, a submerged platform, and an automated video-tracking system. The protocol involved an acclimatization phase, multiple days of acquisition training, and a concluding probe trial. During training sessions, escape latency—defined as the duration from immersion to locating and mounting the hidden platform—was documented. In the probe trial, conducted with the platform removed, spatial memory retention was comprehensively evaluated by measuring the time spent in the target quadrant and the frequency of crossings over the former platform location.

### 4.12. Determination of Cerebral Blood Flow

Real-time, in vivo monitoring of cortical cerebral blood flow in rats was executed using a laser speckle contrast imaging system. Following anesthesia induction with sodium pentobarbital, cranial fur was shaved, and a midline incision was performed along the sagittal suture to expose the skull. Connective tissues were meticulously cleared to ensure optimal imaging conditions. The cranial surface was maintained at a depth of 76 units, while core body temperature was strictly regulated at 37.0 ± 0.2 °C. Throughout the imaging procedure, the detection probe was positioned approximately 10–12 cm superior to the surgically prepared cranial window. Raw speckle contrast data, derived from light scattering within the targeted cerebral blood flow region, were processed using PimSoft V1.4 software to generate cerebral perfusion maps and compile experimental datasets.

### 4.13. Evaluation of Cerebral Infarction Volume

Post-ischemia/reperfusion (I/R) infarct volumes in rats were quantified via 2,3,5-triphenyltetrazolium chloride (TTC) staining. Twenty-four hours following model induction, brains were harvested and serially sectioned into five coronal slices, each approximately 2 mm thick. These sections were incubated in a 2% TTC solution at 37 °C in darkness for 20 min, followed by fixation in 4% paraformaldehyde. Viable tissue exhibited red staining, whereas infarcted regions remained pale. Section images were analyzed using ImageJ software (Rasband, W.S., ImageJ, U. S. National Institutes of Health, https://imagej.net/ij/); infarct volume was calculated based on the volumetric disparity between hemispheres and expressed as a relative percentage.

### 4.14. Histopathological Examination

Following model establishment, rat brains were excised and fixed in 4% paraformaldehyde prior to paraffin embedding. Tissue sections were cut to a thickness of 3–5 μm and stained with either hematoxylin and eosin (H&E) or toluidine blue. Stained sections were evaluated under a light microscope, with representative fields selected for imaging and subsequent analysis.

Histopathological injury score evaluation: The semi-quantitative scoring system consists of three components: “liquefactive necrosis,” “inflammatory cells,” and “red neurons.” Liquefactive necrosis represents the end-stage manifestation of the ischemic infarct core, marking complete tissue death and dissolution. Inflammatory cells (primarily neutrophils and microglia/macrophages) aggregate as a tissue response to injury; the assessment focuses on the number of inflammatory cells infiltrating perivascular regions and the parenchyma. Red neurons are among the most typical and earliest hallmark morphologies of acute ischemic neuronal necrosis, signifying irreversible neuronal injury; the assessment evaluates the proportion of red neurons present in the field of view. Each component is scored on a scale from 0 to 4, with higher scores indicating more severe tissue damage. The three components are evaluated separately, and then a comprehensive score is calculated. References to the Supplementary Tables S2–S4 are provided for the detailed criteria. The text explicitly states that all behavioral and histopathological assessments were performed by investigators blinded to the experimental groups.

### 4.15. Immunohistochemistry Staining Assay

After deparaffinization, brain sections underwent antigen retrieval by heating in 3% citrate buffer (pH 6.0) using a microwave until boiling, then maintaining a sub-boiling temperature for 10–15 min. Sections were blocked with 10% normal serum at room temperature for 1 h, followed by overnight incubation with primary antibodies at 4 °C. Subsequently, secondary antibodies were applied and incubated for 1 h at room temperature. Following development with diaminobenzidine and counterstaining with hematoxylin, sections were examined and imaged via light microscopy.

### 4.16. Expression and Purification of NAMPT Recombinant Protein in a Prokaryotic System

The prokaryotic His-NAMPT-pET28a(+) plasmid was constructed by Gene Create Biological Engineering Co. (Wuhan, China), which also supplied the *Escherichia coli* strains. Sequencing verified successful plasmid construction. Initially, the target plasmid was transformed into Rosetta (DE3) competent cells, incubated on ice for 30 min, and subjected to heat shock at 42 °C for 90 s. After recovery, cells were plated on LB agar containing kanamycin and incubated at 37 °C overnight to isolate positive single colonies. A single colony was then inoculated into LB liquid medium supplemented with kanamycin and cultured at 37 °C until the optical density at 600 nm (OD600) reached approximately 0.6. Isopropyl β-D-1-thiogalactopyranoside (IPTG) was added to a final concentration of 0.1 mM, and induction proceeded at 37 °C for 4 h. Cells were harvested, lysed, and analyzed by SDS-PAGE. Subsequently, the culture was scaled up at a 1:1000 ratio until OD600 ≈ 0.6. The temperature was lowered to 16 °C, and induction was performed with 0.1 mM IPTG for 8 h. Cells were collected via centrifugation, resuspended in pre-cooled NTA-0 buffer, treated with lysozyme, and disrupted by sonication. Following centrifugation, the supernatant and pellet fractions were isolated and analyzed via SDS-PAGE. Subsequently, the supernatant was filtered through a 0.22 μm membrane and applied to a Ni-NTA column at a flow rate of 1 mL/min. The column underwent sequential washing with NTA-0 buffer, followed by gradient elution using buffers supplemented with 20, 60, 200, and 500 mM imidazole. Collected eluates were dialyzed, concentrated, and their purity verified by SDS-PAGE.

### 4.17. LC-MS/MS Analysis of Protein S-Sulfhydration by NaHS and NAMPT In Vitro

#### 4.17.1. S-Sulfhydration-Based Assay for Detecting S-Sulfhydration NAMPT

Briefly, 25 μL of NAMPT protein (50 μg) was mixed with 0, 0.25 μM, 2.5 μM, or 25 μM NaHS in reaction buffer to a final volume of 50 μL. The mixture was incubated with shaking at room temperature for 1 h, and the reaction was terminated by adding 10 mM iodoacetamide. The samples were then sent to Hangzhou Baigerui Biotechnology Co., Ltd. for detection of sulfhydryl modification of NAMPT using LC-MS/MS.

#### 4.17.2. Detection of S-Sulfhydration Modification of NAMPT by LC-MS/MS

Protein Digestion and Peptide Preparation

Proteolytic digestion was conducted using 100 μg of protein solution. Samples were initially reduced with 50 mM DTT at 37 °C for 1 h, followed by alkylation with 100 mM iodoacetamide (IAM) in the dark at room temperature for 40 min. The treated samples were then desalted and buffer-exchanged utilizing a 10 kDa molecular weight cutoff ultrafiltration device. This device was washed twice with 8 M urea (pH 8.5) and three times with 25 mM ammonium bicarbonate (NH_4_HCO_3_). After transferring the device to a fresh collection tube, trypsin was added at an enzyme-to-substrate ratio of 1:50 (*w*/*w*) in 25 mM NH_4_HCO_3_ (total volume 50 μL) and incubated overnight at 37 °C. On the following day, additional trypsin was introduced at a 1:100 (*w*/*w*) ratio, and digestion proceeded for another 4 h at 37 °C. Post-digestion, peptides were recovered by centrifugation, and the filter was rinsed once with 50 μL of 25 mM NH_4_HCO_3_. The filtrates were pooled, yielding approximately 100 μL of peptide solution.

Peptide Desalting

Desalting of peptides was performed using ZipTip C18 pipette tips. Tips were equilibrated with 50 μL of 60% acetonitrile (ACN)/0.1% trifluoroacetic acid (TFA), followed by 10 μL of 0.1% TFA. The peptide sample was aspirated and dispensed 20 times to ensure binding. The tip was then washed five times with 10 μL of 0.1% TFA. Finally, peptides were eluted with 10 μL of 60% ACN/0.1% TFA into a new tube and dried via vacuum centrifugation.

Liquid Chromatography-Tandem Mass Spectrometry (LC-MS/MS) Analysis

Dried peptides were reconstituted in 20 μL of 0.1% formic acid (FA) solution, vortexed, and centrifuged at 17,000× *g* for 20 min at 4 °C. The supernatant was collected, and 3 μL was injected for LC-MS/MS analysis. Peptide separation was achieved on a reversed-phase C18 column employing the following mobile phases: A, 0.1% FA in water; B, 0.1% FA in acetonitrile. The chromatographic gradient was executed at a constant flow rate of 250 nL/min over 70 min according to the following program: 0–8 min, 5–10% B; 8–33 min, 10–15% B; 33–43 min, 15–28% B; 43–50 min, 28–40% B; 50–60 min, 40–95% B; 60–65 min, 95% B; followed by re-equilibration to 5% B from 65 to 70 min. Mass spectrometry was conducted in data-dependent acquisition mode. Full-scan MS1 spectra were recorded in Data-Dependent Acquisition (DDA) mode at a resolution of 70,000 across an *m*/*z* range of 300–1400, utilizing an automatic gain control (AGC) target of 3 × 10^6^ and a maximum injection time of 60 ms. The 20 most abundant precursor ions were sequentially isolated for fragmentation. Corresponding MS2 spectra were acquired at a resolution of 17,500, with an AGC target set to 5 × 10^4^ and a maximum injection time of 80 ms. Fragmentation was induced using a normalized collision energy (NCE) of 27. Subsequent database searches were executed employing PEAKS software (Version 12.5).

### 4.18. Statistical Analysis

All experimental results are expressed as mean ± standard deviation (SD). Statistical evaluations were conducted using GraphPad Prism version 8.0 (GraphPad Software, La Jolla, CA, USA). The difference between the two groups was analyzed using *t*-test. Differences among multiple groups were assessed via one-way analysis of variance (ANOVA) followed by Tukey’s post hoc test, with statistical significance defined as a *p*-value < 0.05.

## 5. Conclusions

This study demonstrates, for the first time, that hydrogen sulfide (H_2_S) confers protection against cerebral ischemia/reperfusion (I/R) injury in rats through S-sulfhydration of NAMPT, the rate-limiting enzyme governing NAD^+^ biosynthesis. This post-translational modification enhances mitochondrial functionality and promotes autophagy within cerebrovascular endothelial cells (ECs). These findings elucidate a novel neuroprotective mechanism wherein H_2_S modulates the central hub of cellular energy metabolism via the H_2_S-NAMPT-NAD^+^ axis. Furthermore, this pathway represents a promising therapeutic target for the development of next-generation neuroprotective agents.

## Data Availability

The original contributions presented in this study are included in the article/Supplementary Material. Further inquiries can be directed to the corresponding authors.

## Supplementary Materials

The following supporting information can be downloaded at https://www.mdpi.com/article/10.3390/ph19050742/s1, Table S1. Rats’ Neurological Deficit Score (Zea-Longa), Table S2. H-E staining injury score (Liquefactive Necrosis Score), Table S3. H-E staining injury score (Inflammatory Cell Infiltration Score), Table S4. H-E staining injury score (Red Neuron Score), Figure S1. Identification of NAMPT prokaryotic plasmid.

## Abbreviations

The following abbreviations are used in this manuscript:

ECs	Endothelial Cells
H/R	Hypoxia/Reoxygenation
I/R	Ischemia–Reperfusion
LC-MS/MS	Liquid Chromatography-tandem Mass Spectrometry
LDH	Lactate Dehydrogenase
MCAO/R	Middle Cerebral Artery Occlusion/Reperfusion
NAMPT	Nicotinamide Phosphoribosyltransferase
NAD	Nicotinamide Adenine Dinucleotide
NSE	Neuron-Specific Enolase
TEM	Transmission Electron Microscopy
