# Supplementary material for: Hydrogen Sulfide Protects Against Cerebral Ischemia–Reperfusion Injury in Rats via S-Sulfhydrating NAMPT to Enhance Mitochondrial Function and Autophagy in Cerebrovascular Endothelial Cells
Source: Pharmaceuticals (Basel). 2026 May 8;19(5):742. doi: 10.3390/ph19050742 (PMC13209901; doi:10.3390/ph19050742)
Supplement: Supplementary file 1 [file pharmaceuticals-19-00742-s001.zip › pharmaceuticals-4237360-supplementary.pdf]

Hydrogen sulfide protects against cerebral ischemia-reperfusion injury in rats via s-sulfhydrating NAMPT to enhance mitochondrial function and autophagy in cerebrovascular endothelial cells

La Jiang[1], Shuai Liang[1], Yu Jiang[1], Jia-Rong Jiang[1],Shan Wang[1], Xiaojiao Yin[1], Zhiwu Chen[1,2*], Jiyue Wen[1*], Shuo Chen[3]; *; 2346010032@stu.ahmu.edu.cn (L.J.); 2345010187@stu.ahmu.edu.cn (S.L.); 2001500028@ahum.edu.cn (J.-Y.W.)

[1]Department of Pharmacology, School of Pharmaceutical Sciences, Anhui Medical University, Hefei 230032, China
[2]Clinical Medical College, Anhui Medical University, Hefei 230012, China
[3]Key Laboratory of Xin'An Medicine, Ministry of Education, Anhui University of Chinese Medicine, Hefei 230038, China

[*]**Correspondence**:

chenzhiwu@ahmu.edu.cn (Z.C.)

chasemoon@aliyun.edu.cn (S.C.);

Supplementary Table S1. Rats' Neurological Deficit Score (Zea-Longa)

Score	Symptom description	Degree of neurological deficit
0	No neurological deficits	Normal
1	Mild: inability to fully extend the contralateral forelimb during tail lifting	Mild defect
2	Moderate: Rotate in circles to the opposite side while walking	Moderate defect
3	Severe: tilting towards the opposite side while walking	Severe defects
4	Unable to walk spontaneously, loss of consciousness	Extremely severe/near death

Supplementary Table S2. H-E staining injury score (Liquefactive Necrosis Score)

Score	Description	Appearance under microscope
0	No necrosis	The organizational structure is complete, with no voids or areas of cell loss.
1	Minimal focal necrosis	Occasionally, there is a very small range of cell loss and vacuolization, with an area of less than 5%.
2	Multifocal/Mild Necrosis	Multiple small focal necrotic areas, or a single smaller necrotic lesion, accounting for a total area of 5% -25%.
3	Moderate fusion necrosis	The necrotic area merges into patches, forming a large range of cavities, and most of the normal structures disappear, accounting for 25% -50% of the area.
4	Extensive necrosis	The vast majority of this brain region has been replaced by necrotic tissue, forming a huge cavity, and normal structures have completely disappeared, with an area greater than 50%.

Supplementary Table S3. H-E staining injury score (Inflammatory Cell Infiltration Score)

Score	Description	Appearance under microscope
0	No infiltration	The space around the blood vessels is clear, and no inflammatory cells are found in the brain parenchyma.
1	Mild infiltration	Occasionally, there is a small amount of inflammatory cell sleeve like infiltration around the blood vessels (about 5-15 per high-power field of view [HPF]), and scattered cells are occasionally seen within the parenchyma.
2	Moderate infiltration	There are more obvious inflammatory cell sleeves around the blood vessels (about 15-30 per HPF), and multifocal inflammatory cell aggregation can be seen in the parenchyma.
3	Severe infiltration	Widespread perivascular inflammatory cell sleeve, increased thickness (about 30-50 per HPF), with a large number of diffuse or clustered inflammatory cells distributed in the parenchyma.
4	Extremely severe infiltration	The blood vessels and parenchyma are filled with dense inflammatory cells, almost replacing normal brain parenchymal cells (>50 per HPF).

Supplementary Table S4. H-E staining injury score (Red Neuron Score)

Score	Description	Appearance under microscope
0	No red neurons present	All neurons have normal morphology, with large and round nuclei, clear nucleoli, and abundant Nissl bodies in the cytoplasm (alkaline, light blue).
1	A very small number of red neurons	Occasionally scattered red neurons (accounting for less than 5% of all neurons) are observed.
2	A small amount of red neurons	Multiple red neurons can be seen (accounting for 5% -25%).
3	A large number of red neurons	Red neurons are widely present and become the main cell type (accounting for 25% -50%). Normal neurons are significantly reduced.
4	Diffuse red neurons	Almost all neurons within the field of view appear as red neurons (>50%), and normal neurons almost disappear.

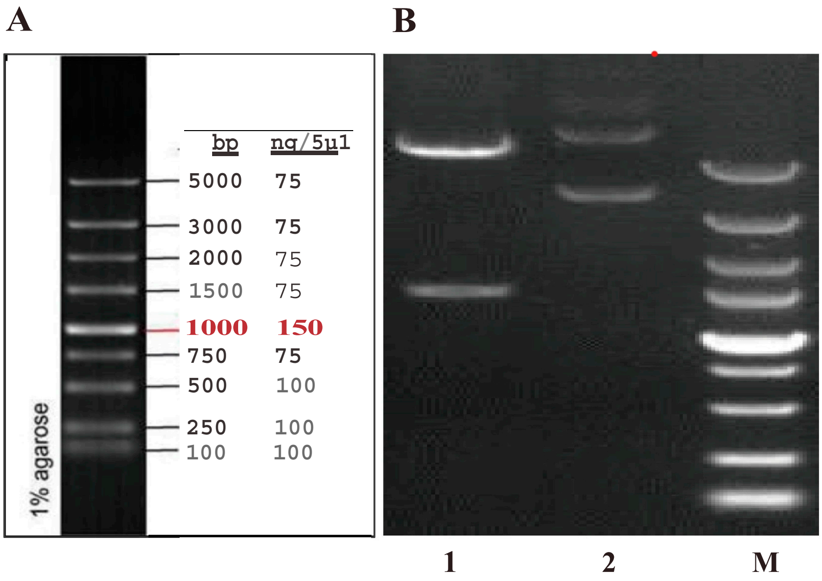

Supplementary Fiure S1 NAMPT-pET28a (+) identification of prokaryotic plasmids.
(A).Restriction map.
(B).Lane M: DNA Marker; Lane **l:** Plasmid digested by BamH,I Xhol; Lane 2: Plasmid DNA.
